# Mobile Health to Improve Adherence and Patient Experience in Heart Transplantation Recipients: The mHeart Trial

**DOI:** 10.3390/healthcare9040463

**Published:** 2021-04-14

**Authors:** Mar Gomis-Pastor, Sonia Mirabet Perez, Eulalia Roig Minguell, Vicenç Brossa Loidi, Laura Lopez Lopez, Sandra Ros Abarca, Elisabeth Galvez Tugas, Núria Mas-Malagarriga, Mª Antonia Mangues Bafalluy

**Affiliations:** 1 Pharmacy Department, Hospital de la Santa Creu i Sant Pau, IIB Sant Pau, 08025 Barcelona, Catalonia, Spain; 2 Cardiology Department, Hospital de la Santa Creu i Santa Pau and CIBER de Enfermedades Cardiovasculares (CIBER-CV), 08041 Barcelona, Catalonia, Spain; smirabet@santpau.cat; 3 Heart Failure and Heart Transplant Unit, Cardiology Department, Hospital de la Santa Creu i Santa Pau, 08041 Barcelona, Catalonia, Spain; eroigminguell@gmail.com (E.R.M.); vbrossa@santpau.cat (V.B.L.); llopezl@santpau.cat (L.L.L.); sros@santpau.cat (S.R.A.); egalvez@santpau.cat (E.G.T.); 4 Pharmacy Department, Hospital de la Santa Creu i Santa Pau, 08025 Barcelona, Catalonia, Spain; nmasm@santpau.cat; 5 Pharmacy Department, Hospital de la Santa Creu i Santa Pau and CIBER de Bioingeniería, Biomateriales y Nanomedicina (CIBER-BBN), 08025 Barcelona, Catalonia, Spain; mmangues@santpau.cat

**Keywords:** heart transplantation, medication therapy management, immunosuppression, treatment outcome, interdisciplinary health team, patient-reported outcome measures, behavioral sciences, treatment adherence and compliance, telemedicine, mobile health

## Abstract

Non-adherence after heart transplantation (HTx) is a significant problem. The main objective of this study was to evaluate if a mHealth strategy is more effective than standard care in improving adherence and patients’ experience in heart transplant recipients. Methods: This was a single-center, randomized controlled trial (RCT) in adult recipients >1.5 years post-HTx. Participants were randomized to standard care (control group) or to the mHeart Strategy (intervention group). For patients randomized to the mHeart strategy, multifaceted theory-based interventions were provided during the study period to optimize therapy management using the mHeart mobile application. Patient experience regarding their medication regimens were evaluated in a face-to-face interview. Medication adherence was assessed by performing self-reported questionnaires. A composite adherence score that included the SMAQ questionnaire, the coefficient of variation of drug levels and missing visits was also reported. **Results**: A total of 134 HTx recipients were randomized (intervention N = 71; control N = 63). Mean follow-up was 1.6 (SD 0.6) years. Improvement in adherence from baseline was significantly higher in the intervention group versus the control group according to the SMAQ questionnaire (85% vs. 46%, OR = 6.7 (2.9; 15.8), *p*-value < 0.001) and the composite score (51% vs. 23%, OR = 0.3 (0.1; 0.6), *p*-value = 0.001). Patients’ experiences with their drug therapy including knowledge of their medication timing intakes (*p*-value = 0.019) and the drug indications or uses that they remembered (*p*-value = 0.003) significantly improved in the intervention versus the control group. **Conclusions**: In our study, the mHealth-based strategy significantly improved adherence and patient beliefs regarding their medication regimens among the HTx population. The mHeart mobile application was used as a feasible tool for providing long-term, tailor-made interventions to HTx recipients to improve the goals assessed.

## 1. Introduction

Lack of adherence to immunosuppressive medication (medication non-adherence, MNA) carries serious risks after heart transplant (HTx) [1,2,3,4,5]. MNA at any time post-HTx is an independent risk factor for acute rejection episodes and cardiac allograft vasculopathy (CAV) over a period of 3–5 years [6]. Moreover, MNA was the key determinant in 90% of late acute rejection episodes (>1 year) and in 13% to 36% of all deaths [6].

The importance of medication adherence has long been established by the transplant community [3,7] and many efforts have been made to reduce MNA, but with little success [8,9]. MNA behavior is a complex and dynamic process that is influenced by several risk factors [10,11]. Some are patient-related, such as patients’ beliefs about their treatment, but this is not the only reason for non-adherence [12,13] and treatment (such as side effects or medication complexity) [6], prescriber and system-related factors may also be involved [3].

A multidimensional approach that takes these multiple factors into account is widely recommended [14]. This includes a combination of proactive strategies [4], such as improving patient information, motivation and skills and improving communication with health providers. In this respect, holistic outpatient programs can have a significantly positive effect on use of medications, lifestyles, quality and efficiency [15,16,17,18]. The use of mobile devices in the health field (mHealth) has enormous potential to transform healthcare and implement patient-centered programs [19,20,21,22,23]. The mHealth application offers a unique opportunity to support the implementation of a new outpatient care program in the transplant population [24].

Furthermore, behavioral change science may offer a better understanding of the origin of patient non-adherence behavior and how the intervention implemented by the professionals works [25]. It also increases the effectiveness of treatments [26] and offers greater comparability and the generalized application of successful interventions [27,28]. Although there is some evidence that behavior change theories can be useful in HTx recipients [29,30], behavioral intervention programs are lacking in this population [1,8,31].

**Figure 1:**
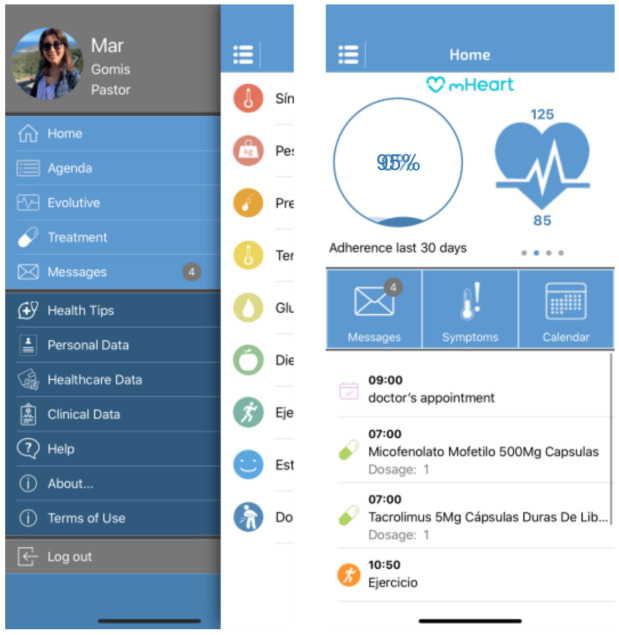
The mHeart mobile application main screen captures. The different app modules are displayed: Treatment, Agenda, Self-reliance, Symptoms, Messaging, Health Education and Advice, Personal and Clinical Data. The platform details have been published in the pilot study [34], a summary is also provided in Supplementary File S1.

**Figure 2:**
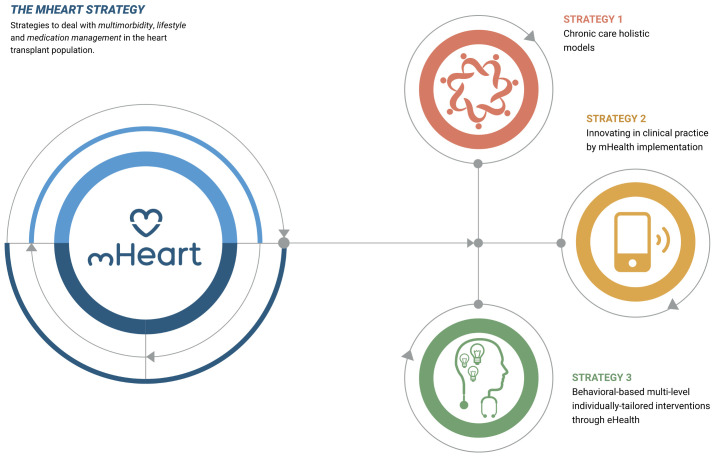
The mHeart strategy components. The strategy definition and design has been published in the pilot study [34], a summary is also provided in Supplementary File S1.

The mHeart tool is a mobile application complemented by a website [32,33]. (Figure 1) It was designed by the multidisciplinary HTx team to support an intensive, individualized, behavior-based strategy to improve the safety and efficacy of medication taking and to facilitate comprehensive care in HTx recipients (Figure 2). A previous pilot study validated the feasibility of this intervention and satisfaction of patients with the mHeart tool [34]. 

Objectives. The main objective of this study was to assess if the mHeart strategy improves medication adherence and patients’ experience with their medication regimens in HTx recipients compared to standard care. The secondary objective was to explore the impact of the mHeart strategy on long-term clinical care and follow-up of HTx recipients.

## 2. Materials and Methods

### 2.1. Study Setting

This was a single center, randomized controlled trial (RCT) comparing standard care with the mHeart strategy in improving adherence in adult HTx recipients. The mHeart tool is a mobile and web-based software application (Figure 1). The main features of the platform are designed to (i) identify MNA recipients, (ii) resolve patients’ doubts about their treatment and health status, (iii) empower patients in terms of self-care and (iv) facilitate professionals’ interventions based on online patient-reported outcomes. The description, development and quality assurance of the software (mHeart Version 3) have been published in a pilot study [34]. Details about its clinical use and functionalities are provided in Supplementary File S1 and Mendeley Dataset [32]. No downtimes occurred and no content changes were made to the system during the study period. Any bugs were resolved by the technical team to enhance the usability of the platform by recipients.

The study was approved by the Institutional Review Board of the hospital (IIBSP-MHE-2014-55) and was registered in Clinicaltrials.gov (ID MHEART: NCT02554578). A pilot study to validate the feasibility of the intervention included patients who were <1.5 years post-transplant [34]. The present study included adult HTx recipients who were >1.5 years post-transplant. Exclusion criteria were severe clinical decompensation rendering interview impossible (physician-based judgment), severe cognitive impairment rendering interview or the use of the software impossible (physician-based judgment), patients with other illnesses such as Parkinson’s disease or severe tremor rendering the use of the software impossible, patients not owning a mobile device and patients living in care centers, limiting their medication self-management. No prior computer or internet literacy was required. Participants did not receive any financial compensation, nor did they receive a phone or wearables for their participation. 

The standard of care in our hospital prior to enrollment in the study consisted of the teaching and supervision of medications by the specialist nurse and cardiologists at the time of discharge and during in-clinic medical appointments. At the time of the study, the clinical pharmacist was included in the multidisciplinary team. To avoid bias, recipients had no previous interaction with the pharmacist prior to the baseline visit. 

### 2.2. Design

**Figure 3:**
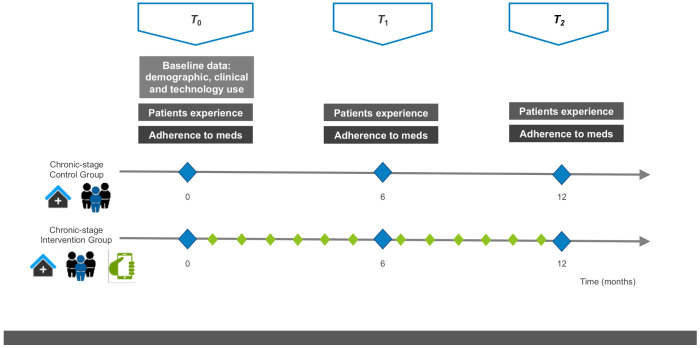
Study design. Scheduled in-clinic visits are shown as triangles: T_0_ (baseline at study inclusion), T_1_ (at least 6 months after inclusion), T_2_ (at least 12 months after inclusion). The variables assessed during scheduled visits are shown as squares: baseline information, patient experience and medication adherence. Treatments are shown as pictograms, i.e., (i) in-clinic visits at the Hospital outpatient department, (ii) multidisciplinary team including the pharmacist and (iii) the mHeart mobile application for remote interaction with the pharmacist. The diamonds show the scheduled interaction with the clinical pharmacist to perform interventions: blue (during the scheduled in-clinic visits; all patients) and green (using the mHeart tool; intervention group only).

The study design is shown in Figure 3.

Candidates for participation were contacted by telephone by a research assistant prior to study initiation to briefly explain the study and to ask for their agreement to include an additional visit with the clinical pharmacist on the same day as their next scheduled visit. Eligible patients gave written informed consent. They were consecutively enrolled during their scheduled visit by the clinical pharmacist (T0). At the end of the baseline visit patients were randomly assigned at a ratio of 1:1 to the control group (CG) or the intervention group (IG). Baseline measures and counseling by the pharmacist to improve medication self-management were performed before the allocation was known. For all patients included in the study, two more visits were scheduled *T*_1_ (at least 6 months after inclusion) and *T*_2_ (at least 12 months after inclusion). 

At *T*_0,_ *T*_1_ and *T*_2_ all patients received counseling by the pharmacist on how to improve medication self-management using behavioral theory-based treatments [35]. These interventions were delivered using motivational interviewing [36,37], which is recognized as a common practice pattern to improve post-transplant medication adherence in HTx centers [15]. Optimization of medical treatment was also performed in order to reduce therapeutic complexity. Additionally, for patients randomized to the mHeart strategy (IG), multifaceted theory-based interventions were provided during the study period to optimize therapy management using the mHeart tool. The mHeart intervention procedures and algorithm have been validated previously in the pilot study [34] and has been detailed in Supplementary File S1. 

In-clinic visits were conducted according to a fixed template and the information was recorded in the patient’s EHR immediately after the end of the visit by the clinical pharmacist. All data obtained were recorded at the online database Clinapsis [38]. A retrospective review of the EHR and the Clinapsis records was performed by 2 independent research assistants at the end of the study.

### 2.3. Measures

#### 2.3.1. Adherence to Medication Measures

Adherence to medication was evaluated according to the extent to which a patient’s actual dosing corresponded to the prescribed dosing regimen (i.e., omissions of single or consecutive doses, delays in medication taking, or self-initiated dose changes, such as a reduction or increase in dosing, are considered non-adherence). The poor regularity of immunosuppressive treatment refers to dosing discrepancies of ± 2 h [11,39]. Non-persistence was defined as the early discontinuation of the medication [40]. Adherence was recorded at *T*_0,_ *T*_1_ and *T*_2,_ combining qualitative and quantitative methods. These methods were selected to facilitate generalizability to large populations and simplicity in use and scoring.

Three questionnaires were used as qualitative methods to measure adherence to immunosuppressive treatment: the Spanish Version of the SMAQ (Simplified Medication Adherence Questionnaire) [41], the IMTS (Immunosuppressive Medication Timing Scale) and the BAASIS (Basel Assessment of Adherence to Immunosuppressive Medications Scale) [42]. The SMAQ and the IMTS were performed during the interview by the pharmacist and the BAASIS was patient reported. The SMAQ questionnaire is a 6-item scale validated in transplant population that measures patients’ medication habits [41]. The IMTS is a 2-item self-reported, semi-quantitative questionnaire created for the study. We asked the patients about how often they modified their immunosuppressant timetable in the last week and since the last scheduled visit. If the answer was “Never” for both questions, the patient is considered to be adherent. The BAASIS has been validated in transplant recipients and measures patients’ medication taking, omission, timing and dose reduction of immunosuppressive medication [42]. BAASIS includes the visual analog scale (VAS), which is an overall score ranging from 0 (never took medications as prescribed) to 100 (always took medications as prescribed). Adherence to other co-medications was assessed at *T*_0,_ *T*_1_ and *T*_2_ visits, using the Haynes-Sackett questionnaire (Spanish version) [43]. This is a 1-item scale that asks patients the question: “Most patients have difficulty taking all their tablets, do you have difficulties taking all of yours?” A patient is considered to be non-adherent if he or she responds affirmatively to the question.

Quantitative methods to measure adherence included immunosuppressive medication blood levels and compliance with visits. Variability for tacrolimus and cyclosporine blood levels was assessed by the coefficient of variation of concentrations (CV% = (SD/µ) × 100) and the standard deviation (SD) for each patient. Patients with SD > 2.5 or with CV% > 30% were interpreted as non-adherent [11]. Trough blood levels (ng/mL) were assessed using Fluorescence Polarization Immunoassay at *T*_0_, *T*_1_ and *T*_2._ The therapeutic range for each drug was the range recommended in the ISHLT guidelines [44]. The number and percentage of patients with missing visits at *T*_0_, *T*_1_ and *T*_2_ were recorded by retrospective review of hospital electronic health records (EHR). The SMAQ score at *T*_0_, *T*_1_ and *T*_2_, compliance with the scheduled visits and global CV% were combined to develop a composite adherence score. If any of the 3 variables reflected MNA, the patient was classified as non-adherent. 

#### 2.3.2. Patients’ Experience with Their Medication Regimen

Patients were asked to report their self-reliance for medication management at *T*_0_, *T*_1_ and *T*_2_; the perceived inconvenience of their medication regimens (scored 1 to 10); feeling of taking excessive medication; their opinion of the importance of immunosuppressive treatment and consequences of not taking it; and knowledge of their immunosuppressive regimen; and reported medication side effects.

#### 2.3.3. Type of Pharmaceutical Care Follow-Up at the End of the Study and Beyond

At the end of the study (*T*_2_), patients were categorized based on their future need for face-to-face routine care with the pharmacist versus an online follow-up. The decision was made in a consensus-based manner by the HTx team depending on patient’s self-reliance with regimen management and the medication adherence figures achieved.

#### 2.3.4. Sociodemographic and Clinical Data

Sociodemographic and clinical data were collected at *T*_0_ from the EHR and personal interviews with the pharmacist. Patients’ access to technology and willingness to use mHealth services were collected at *T*_0_ from a questionnaire based on McGillicuddy et al. [45]. Patients using mHeart during the study were categorized according to their use of the tool at the end of the study (*T*_2_). All variables are specified in Supplementary File S2.

### 2.4. Study Reporting Guidelines

We followed the recommended criteria of the ESPACOMP (European Society for Patient Adherence, COMpliance and Persistence) Medication Adherence Reporting Guideline (EMERGE) [40] for transparent and accurate reporting of data on medication adherence. The directions of the ISRII [27] and the CONSORT-EHEALTH guidelines [28] were followed to report the mobile-based intervention and the RCT. The Theory Coding Scheme (TCS) [46] provided a reliable method to describe the theory underpinning the interventions.

### 2.5. Statistical Analysis

The sample size was calculated to detect a difference in adherence measured with the SMAQ scale between *T*_0_ and *T*_2_ of at least 25%. The statistical power was 80% using a 2-tailed test run at an alpha level of 0.05. The resulting sample size was 136 patients (1:1 allocation) including dropouts or losses to follow-up (estimating at least a 10% loss).

For descriptive statistics, categorical variables are expressed as the number of cases (N) and their percentage (%), while quantitative variables are expressed as mean (M) and standard deviation (SD). Ordinal or quantitative variables with non-normal distribution are expressed as median (ME) and quartiles 25-50-75 (IQR). 

Contrast analysis of the IG and CG groups was conducted and the improvement in each study group at times between *T*_0_ and *T*_2_ was compared. The analysis included parametric tests (t-test) and non-parametric tests (Mann–Whitney) for continuous variables (depending on the normality of the distribution, the Kolmogorov–Smirnov or Shapiro–Wilk test was used) and Chi-squared tests or Fisher’s exact test, as appropriate, for the remaining categorical variables. The results of comparisons are described by odds ratios (OR) with their corresponding 95% confidence interval (95% CI) for categorical variables or the magnitude of the difference for quantitative variables, as well as the statistical significance (*p*-value) of the difference. ORs were not calculated for polychotomous variables (those with more than 2 distinct categories). For all analyses, statistical significance was set at 5% (α < 0.05) with 80% power (β = 0.20). All statistical tests were 2-tailed. Missing values were not imputed nor were anomalous values substituted. For some values, the between-group differences were significant (*p*-value > 0.05), but OR and 95% CI could not be calculated due to the lack of information on one or more of the categories of the variable (zero cases). In these cases, the magnitude of the difference and its precision are unknown.

The statistical analysis was performed with IBM-SPSS (V25.0) and R version 3.5.2 by an independent statistician.

## 3. Results

**Figure 4:**
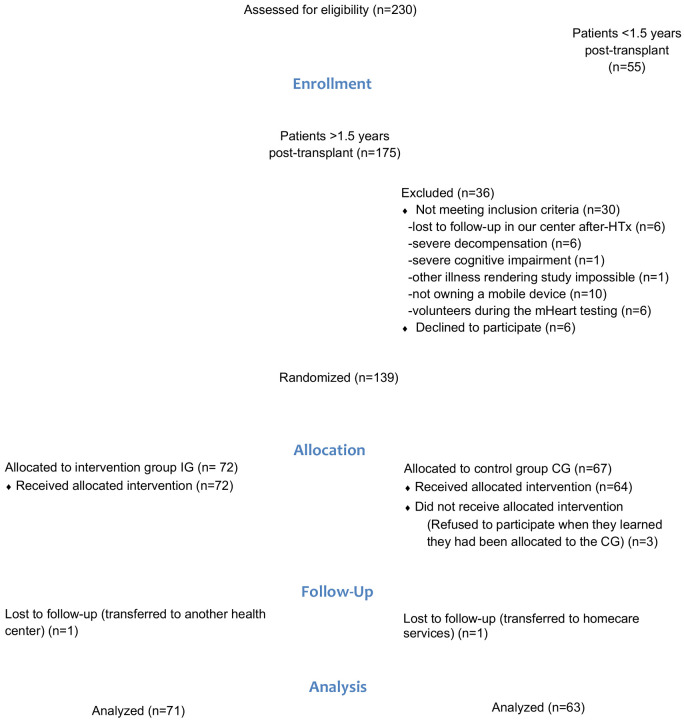
Patient flow chart according to the CONSORT guidelines.

Between 15 July 2015 and 31 December 2018, a total of 220 HTx recipients were evaluated and 134 HTx recipients were randomized (IG N = 71; CG N = 63). An attrition rate of 4% was observed (Patient flow chart Figure 4).

Mean follow-up after inclusion in the study was 1.6 (SD 0.6) years. Demographic and clinical characteristics are summarized in Table 1 and Supplementary File S3. No statistically significant differences were found in baseline variables between groups except for employment status (*p*-value = 0.038). Mean age was 57 (SD 14) years; 31% were women. Mean time from HTx was 11 (SD 7) years. The mean total medication count was 10 drugs (SD 3, range 3–18) and most patients were treated with tacrolimus (73%), mycophenolate (75%) and prednisone (85%). With respect to lifestyle habits (data not shown) in the last 3 months, 32% of the patients reported not practicing any sport, 8% had smoked, 73% reported alcohol consumption (36% > 3 times/week) and 3% reported drug consumption.

At baseline, 65% of the recipients reported that they used technology frequently. Up to 63% used the internet to seek health information. A total of 72% of the patients reported that a tool such as mHeart could be “useful” or “very useful”. A quarter (24%) of the patients reported they might need help to get started using a tool like the mHeart platform. At the end of the study, 85% of the patients assigned to mHeart were engaged on a daily basis, but 10% of them needed to be reminded at least once during the study period to use the mHeart platform. None of the participants completely stopped using mHeart. (Supplementary File S4)

**Figure 5:**
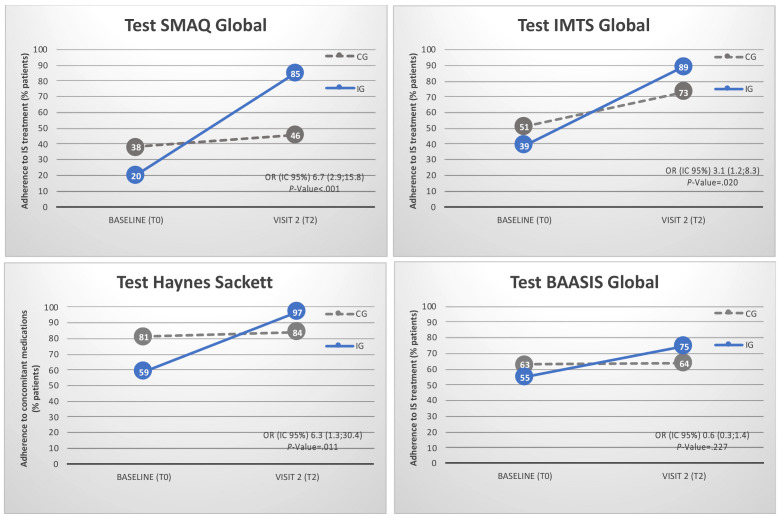
Adherence to immunosuppressive treatment according to the SMAQ interview questionnaire, the Timing of medication taking (IMTS test), the Global BAASIS patient self-reported questionnaire and the Haynes–Sackett adherence to concomitant medication scale. Scheduled in-clinic visits were T0 (baseline at study inclusion) and T2 (at least 12 months after inclusion). Control Group (CG) was based on (i) in-clinic visits at the Hospital outpatient department and (ii) multidisciplinary team including the pharmacist. Intervention Group (IG, the mHeart Strategy) was based on (i) in-clinic visits at the Hospital outpatient department, (ii) multidisciplinary team including the pharmacist and (iii) the mHeart mobile application for remote interaction with the pharmacist.

Adherence to medication is described in Appendix A. Rates by item are specified in Supplementary File S5. Before randomization, only 29% of recipients were completely adherent to immunosuppressive treatment according to the SMAQ interview questionnaire. The BAASIS patient self-reported questionnaire showed an adherence rate of 59%. The Haynes-Sackett scale showed that 69% of recipients were adherent to concomitant medications. At the end of the study (Figure 5), medication adherence rates had significantly improved in the IG (85%) compared with the CG (46%), according to the SMAQ questionnaire (OR = 6.7 (2.9; 15.8), *p*-value < 0.001). Timing of medication taking (IMTS test) also significantly improved in the IG (89%) compared with the CG (73%) (OR = 3.1 (1.2; 8.3), *p*-value = 0.020). The Global BAASIS patient self-reported questionnaire showed a tendency toward improvement in the IG (OR = 6.2 (1.4; 27.9), *p*-value = 0.057). The VAS score (BAASIS) for patients’ self-reported feeling of adequate therapy management significantly improved in the IG (*p*-value = 0.033). The Haynes–Sackett scale showed a significant improvement in concomitant medication adherence in the IG (97%) compared with the CG (84%) (OR = 0.3 (0.1; 0.6), *p-*value = 0.001).

A CV > 30% was observed in 49% of recipients (47% CG; 41% IG, *p*-value = 0.526). The mean number of therapeutic blood levels (*p*-value < 0.001) and the mean number of supratherapeutic concentrations were significantly improved in the IG (*p*-value = 0.050). According to the composite adherence score, overall adherence was significantly improved in the IG (51%) versus the CG (23%) (OR = 0.3 (0.1; 0.6), *p*-value = 0.001).

Patient experience measures are shown in Appendix B and detailed by item in Supplementary File S6. Medication-related inconvenience reduced significantly in the IG (0.5/10, SD 2) compared with the CG (2/10, SD 3) at the end of the study (*p*-value = 0.002). As many as 63% of recipients believed they were taking excessive medication at baseline (*T*_0_), while these percentages had reduced significantly in the IG at *T*_2_ (69% vs. 36%, *p*-value < 0.001). As many as 28% of patients reported that they were unaware of the consequences of discontinuing immunosuppressive therapy and 41% were unaware of the consequences of sometimes forgetting to take their immunosuppressive treatment. These rates had improved significantly at the end of the study in both groups (*p*-value < 0.02). Patients remembered 76% of the names of their medications (brand name or active ingredient), 51% of doses, 79% of timing taking and 62% of the drug indications or drug uses to treat at baseline (*T*_0_). All these rates had significantly improved at the end of the study (*T*_2_) in the IG compared with the CG (*p*-value < 0.03). The mean number of side effects reported by patients at baseline was 6 (SD 3). This number fell significantly to 3 (SD 2) at the end of the study in both groups (*p*-value < 0.001). 

A mean of 3 (SD 1) personalized in-clinic interventions to improve patients’ medication self-management were performed by the pharmacist during scheduled visits. The most frequent were to monitor interactions (78% of recipients), recommend the use of a pillbox (60%) and optimize treatment to reduce medication complexity on at least 1 occasion (71% of the recipients). Supplementary File S7: Medication complexity management had improved at the end of the study (*T*_2_) in the IG since the total number of drugs to treat comorbidities (*p*-value < 0.001) and the number of over-the-counter medications (OTC) (*p*-value = 0.063) had reduced. The proportion of patients who would need to continue in-clinic visits with the pharmacist after the study ended was 35% in the IG and 65% in the CG (OR = 3.4 (1.7;6.9), *p*-value = 0.001). (Table 2)

## 4. Discussion

The results of this study indicate that the mHeart tool is significantly more effective in improving adherence than standard care in HTx recipients. An increase of 65% in adherence was reported in the intervention group compared to 8% in the control group. Non-adherence is a significant problem among HTx recipients and concerns have arisen about its consequences [1,2,3,4,5]. Previous studies reported a non-adherence rate of 15–50% in HTx recipients [3,8,13,47], but conventional interventions have showed limited ability to modify recipients’ behavior [9,10,39]. There is, therefore, an urgent need for innovative strategies to reduce MNA rates in a digitalized society [8,15,39].

This is the first mHealth tool that has been shown to improve adherence in HTx recipients. These results suggest that the strategies used in our study have a synergic effect. In contrast to other studies, our intervention program was designed to deliver personalized internet-based multilevel interventions based on behavioral theories [13,35,48]. Indeed, human support and tailored interventions have been shown to be a requisite to improve MNA rates throughout eHealth [10,49]. Moreover, our pilot study met 72% of the TCS criteria (i.e., items 1–11) [34], indicating that the interventional study design complies with the theoretical basis of the intervention [46]. This is important because interventions meeting a minimum of 60% of the TCS criteria have been found to be highly effective [50].

Second, measuring patient experience was helpful in understanding patients’ weaknesses in medication beliefs and in guiding the eHealth interventions. The objective was to achieve empowered and more self-reliant patients. Easy access to information, good communication with healthcare providers, a reduced appointment burden and better coordination among healthcare processes are some of the main patient demands [51]. This study has provided evidence that humanizing healthcare [52] also improves the clinical outcomes measured. Moreover, an in-depth description of the behavioral change model obtained in this study can provide a better understanding of the causes of patients’ behavior and how the intervention works [25], thereby increasing treatment effectivity, comparability and scalability.

Third, the mHeart software was designed to provide intensive individually tailored interventions with the goal to empower patients in medication self-management. Furthermore, in populations with polypharmacy any therapy management program must include optimization strategies to reduce medication complexity [6]. In a previous study, we observed that most of the medication complexity of our HTx population was driven by drugs to treat comorbidities and OTC medication other than immunosuppression [53]. Taking this into account, an interdisciplinary medication optimization was driven during this study to optimize non-immunosuppression therapies. This intervention could also contribute to improve drug adherence rates, as other studies suggest [54,55,56].

Fourth, the mHeart Strategy has been well accepted by patients, with 83% continuing to be engaged with the mHeart at the end of follow-up. In this sense, the results obtained by Park LG, et al. suggest that, in order for a mobile health study to impact on adherence, a high patient satisfaction with the technology and the interventions designed should be also met [21]. Therefore, our results could be influenced by the effort of the interdisciplinary team in educating and motivating the patient in the technology use, but also because the mHeart Help Center (of the private firm developing the technology) was responsible for the patient training and solving technical problems. Moreover, the clinical pharmacist focused her interventions on empowering the patient with their treatment and illness through the mHeart technology, contributing to patient engagement and satisfaction.

It is of utmost importance to point out that none of the patients randomized to the IG dropped out [57]. These positive results reinforce the feasibility of the intervention workflow and support the generalizability of the mHeart strategy. Once the study ended, the mHeart platform went beyond the research project and was incorporated as part of routine care of our HTx population. In our experience, mHealth enhance pharmacist-patient interactions and the number of pharmaceutical interventions solved per day. Moreover, more than half of the patients in the IG continued the follow-up by the pharmacist on an online basis only. These patients are currently receiving periodically personalized feedback through mHeart in order to maintain improvements in medication adherence and attitude towards their treatments. 

The inclusion of the mHeart platform in clinical practice improves adherence to medical treatment, as we have demonstrated, but also facilitates comprehensive remote routine-care. In this sense, the use of the platform was certainly useful during the COVID-19 pandemic, offering the possibility of providing psychological support, sending information to patients and caregivers and avoiding patients to travel to the hospital. This generalizability of the mHeart strategy demanded a proactive interdisciplinary team properly trained in digital behavioral skills to deliver eHealth interventions, as suggested by other authors [15,36,39,58].

Overall, an mHealth strategy offers a valuable opportunity to expand the benefits of more interactive and modern ways of providing medication management, but also to improve patients’ care and experience of the healthcare system [59,60].

Study limitations: This study has some limitations. First, we observed highly different MNA rates depending on the assessment method. This fact has been noted in previous studies, depending on the method and the definition of the term *medication adherence* used in literature [3,61]. Second, self-reporting measures could under-represent non-adherence figures [11,61,62]. To avoid this bias and since there is no gold standard in adherence measurement in the transplant population [61], we used a reliable methodology in transplant population combining several qualitative and quantitative methods [1,11,63]. Third, the study was not blinded, but subjective preferences were unlikely to mask baseline results since diverse solutions were implemented to mitigate subjective interpretations. Recipients had no previous interaction with the pharmacist prior to the baseline visit and baseline measures and counseling by the pharmacist to improve medication self-management at visit T_0_ were obtained before the allocation was known. Furthermore, in-clinic visits were conducted according to a fixed template and the information was recorded in the patient’s EHR immediately after the end of the visit. A retrospective review of these records by two independent research assistants and an independent statistician was also implemented to mitigate subjective interpretations.

## 5. Conclusions

Non-adherence is one of the main challenges in the HTx population. In our study, the mHeart strategy demonstrated a significant increase in adherence in adult HTx recipients compared with standard care. The mHeart strategy had, in addition, a positive impact on patients’ experience of their drug therapy. The degree of patient-perceived inconvenience and patient knowledge of their medication regimens showed a statistically significant improvement in the intervention group. The tool has been well accepted by patients and decreases the need for patients to attend in-clinic follow-up visits with the pharmacist. The mHealth approach will be a feasible way to provide online tailored interventions for HTx recipients.

## 6. Patents

The Hospital Sant Pau I de la Santa Creu Research Institute owns the legal property of the mHeart national trademark and the intellectual property of the mHeart content. No other patents were generated during the study.

## Figures and Tables

**Table 1:** Demographic and clinical data.

Variables		Total HTx Patients (N = 134)
**Demographic information**		
Recipient gender (male), N (%)		92 (69)
Body mass index (kg/m^2^), M ± SD		27 ± 5
Recipient age at the time of the study (years), M ± SD		57 ± 14
Patients > 75 years old, N (%)		5 (4)
Educational attainment, N (%)	No schooling	15 (11)
	Middle school graduate	58 (43)
	High school graduate	25 (19)
	University graduate	36 (27)
Employment status, N (%) ^1^	Disability	74 (55)
	Currently employed	34 (25)
	Retired	19 (14)
	No previous employment activity	7 (5)
**Clinical variables, transplant-related (continue in**Supplementary File S3)		
Recipient age at HTx (years), M ± SD		45 ± 16
Time from HTx (years), M ± SD		11 ± 7
Urgent HTx, N (%)		33 (25)
Heart failure etiology, N (%)	Coronary/ischemic	35 (26)
	Cardiomyopathy	60 (45)
	Other	84 (47)
	Re-transplant	8 (6)
**Multimorbidity and use of care resources among HTxR included in the study**		
Number of comorbidities Post-HTx, M ± SD ^2^		6 ± 3
Need or requirement for caregiver, N (%)		27 (20)
Lives with someone else, N (%)		114 (88)
**Treatment measures**		
Immunosuppressive treatment, N (%)	Cyclosporine	33 (25)
	Tacrolimus	98 (73)
	Everolimus	20 (15)
	Sirolimus	3 (2)
	Azathioprine	4 (3)
	Mycophenolate mofetil	71 (53)
	Mycophenolate sodium	29 (22)
	Corticosteroids	114 (85)
Total drugs count, M ± SD		10 ± 3
Patients with polypharmacy), N (%)	≥8 drugs	100 (75)
	≥15 drugs	9 (7)
Drugs to treat comorbidities, M ± SD		4 ± 2
OTC medicines, M ± SD		2 ± 1
Complementary therapies, M ± SD		2 ± 1

^1^ No statistically significant difference was found in baseline demographic and clinical variables between the control and intervention group except for employment status (*p*-value = 0.038). ^2^ The category comorbid disease is described in Supplementary File S2. Missing values were not imputed nor were anomalous values substituted. See the statistical analysis section in the manuscript for more detail. Abbreviations: BMI, body mass index; HTx, heart transplantation; HTxR, heart transplant recipients; M, mean; OTC, over-the-counter; RCT, randomized controlled trial; SD, standard deviation.

**Table 2:** Type of pharmaceutical care follow-up beyond the end of the study.

Variables, N (%)		Total HTx Patients (N = 134)		
		CG (N = 63)	IG (N = 71)	*P*-Value
No need for regular face-to-face in-clinic appointments	Total	22 (35)	46 (65)	<0.001 *
	Discharge from in-clinic visits	22 (35)	19 (27)	-
	Discharge with intensive mHeart reminders to track medication adherence	0 (0)	18 (25)	-
	Discharge with mHeart reminders to follow lifestyle habits affecting medication regimens	0 (0)	9 (13)	-
Need for regular face-to-face in-clinic appointments	Total	41 (65)	25 (35)	<0.001 *
	Intensive in-clinic follow-up every 6 months	7 (11)	3 (4)	-
	Annual in-clinic follow-up to reinforce medication adherence	28 (44)	14 (20)	-
	Annual in-clinic follow-up for other medication-related issues	6 (10)	8 (11)	-

See variables and methods section for definitions. See the statistical analysis section in the manuscript for more detail. Missing values were not imputed nor were anomalous values substituted. * Pearson’s Chi-squared test (χ2). Abbreviations: RCT, randomized controlled trial.

## Data Availability

Data supporting reported results can be found at the database Clinapsis (www.clinapsis.com accessed on 13 April 2021). An additional dataset was generated during the study in the online Mendeley dataset providing relevant information about the technology developed for great use of the scientific community: Gomis-Pastor M, Mangues MA, Pellicer V. mHeart—mHealthCare Platform Adapted to the Heart Transplant Population—Technical Specifications and Software Source Code. Mendeley Data. doi:10.17632/yf2dgcfmmh.2.

## Supplementary Materials

The following are available online at https://www.mdpi.com/article/10.3390/healthcare9040463/s1. Supplementary File S1: Description of the mHeart strategy (A) the intervention design, (B) the intervention workflow, (C) the mHeart^®^ features. Supplementary File S2: Study measures collected. Supplementary File S3: Donor and receptor clinical data. Supplementary File S4: Technology-related data. A) Patients’ technology-related usability and preferences at baseline, B) Patients’ engagement with the mHeart mobile application at the end of the study. Supplementary File S5: Adherence to medication rates. Supplementary File S6: Patient-experience outcomes. Supplementary File S7: Prevalence of in-clinic personalized interventions by the pharmacist to improve patients’ medication management.

## Abbreviations

BAASIS	Basel Assessment of Adherence to Immunosuppressive Medications Scale
CG	control group
eHealth	use of information and communication technologies for health
EHR	electronic health records
HTx	heart transplant
IG	intervention group
IMTS	Immunosuppressive Medication Timing Scale
ISHLT	International Society of Heart and Lung Transplantation
mHealth	mobile health
mHeart	a mobile health system for the heart transplant population
MNA	medication non-adherence
RCT	randomized controlled trial
SMAQ	Spanish version of the Simplified Medication Adherence Questionnaire

## Appendix A

**Table A1:** Medication adherence improvement over time (T0 vs. T2) and between the control (CG) and intervention (IG) groups.

Adherence Evaluation	Total HTx Patients (N = 134)	CG (N = 63)	IG (N = 71)	Statistics OR (IC 95%)	*P*-Value
**Adherence to IS treatment**					
**SMAQ Global (Adh.), N (%)**					
• T0	38 (29)	24 (38)	14 (20)	0.4 (0.2;0.9)	**0.028 ***
• T2	81 (67)	25 (46)	56 (85)	6.7 (2.9;15.8)	**<0.001 ***
• Statistics OR (IC 95%)		2.2 (0.7; 6.7)	2.3 (0.3;19.7)		
• *P*-value McNemar test		0.286	**<0.001**		
**IMTS Global (Adh.), N (%)**					
• T0	59 (44)	32 (51)	27 (39)	0.6 (0.3;1.2)	0.157 *
• T2	98 (82)	40 (73)	58 (89)	3.1 (1.2;8.3)	**0.020 ***
• (IC 95%)		3.7 (1.0;13.7)	4.2 (0.5;37.5)		
• *P*-value McNemar test		**0.007**	**<.0001**		
**BAASIS Global (Adh.), N (%)**					
• T0	64 (59)	34 (63)	30 (55)	1.4 (0.7;3.1)	0.372 *
• T2	89 (69)	30 (64)	39 (75)	0.6 (0.3;1.4)	0.227 *
• Statistics OR (IC 95%)		12 (2.6;54.2)	6.2 (1.4;27.9)		
• *P*-value McNemar test		1.0	**0.057**		
**BAASIS (5). VAS Scale, M ± SD**					
• T0	93 ± 14	93 ± 13	93 ± 16	-	0.672 **
• T2	95 ± 8	95 ± 7	96 ± 9	-	0.225 **
• *P*-value Friedman test		0.739	**0.033**		
**Adherence to other medications**					
**Haynes Sackett (Adh), N (%)**					
• T0	93 (69)	52 (81)	41 (59)	0.3 (0.2;0.7)	**0.004 ***
• T2	110 (92)	46 (84)	64 (97)	6.3 (1.3;30.4)	**0.011 ***
• Statistics OR (IC 95%)		1.1 (0.2; 6.6)	1.5 (0.1;24.4)		
• *P*-value McNemar test		0.804	**<0.001**		
**Adherence to visits, N (%)**					
T0	133 (99)	64 (100)	69 (99)	_	0.337 *
T2	121 (90)	55 (86)	66 (94)	0.37 (0.1;1.3)	0.103 *
*P*-value McNemar test		-	0.375		
**IS drugs levels**					
**CV%, M ± SD)**	33 ± 18	34 ± 22	29 ± 15	-	0.392 **
**CV% > 30%, N (%)**	87 (49)	30 (47)	29 (41)	-	0.526 ***
**Subtherapeutic blood levels,** **N (M ± SD)**	126 (3 ± 3)	48 (4 ± 4)	49 (3 ± 2)	-	0.251 **
**Supratherapeutic blood levels, N (M ± SD)**	83 (4 ± 4)	23 (4 ± 4)	20 (2 ± 4)	-	**0.050 ****
**Therapeutic blood levels, N (%)**	25 (14)	11 (17)	13 (19)	-	**<0.001 *****
**Composite adherence score ^1^, N (%)**					
T0	27 (15)	13 (20)	6 (9)	-	0.052 ***
T2	60 (34)	15 (23)	36 (51)	0.3 (0.1;0.6)	**0.001 *****
*P*-value McNemar test		0.791	**<0.001**		

^1^ If any of the 3 variables (SMAQ, compliance with the scheduled visits and global CV%) reflected nonadherence to medication, the patient was classified as non-adherent. See variables and methods section for definitions. Missing values were not imputed nor were anomalous values substituted. See the statistical analysis section in the manuscript for more detail. Measurement points: *T*0 (baseline at inclusion into study), *T*2 (at least after 12 months from inclusion). Abbreviations: Adh., adherence to medication; Basel Assessment of Adherence to Immunosuppressive Medications Scale (BAASIS); GI, RCT intervention group; CG, RCT control group; CV, Coefficient of Variability; HTx, heart transplantation; IS, immunosuppressive medication; IMTS, Immunosuppressive Medication Timing Scale; M, mean; Nonadh., Nonadherence to medication; OR, Odds Ratio; RCT, Randomized controlled trial; SMAQ, Simplified Medication Adherence Questionnaire; SD, standard deviation; VAS, visual analog scale. * Pearson’s Chi-squared test (χ2); ** Mann-Whitney U-test (exact sig 2-tailed); *** χ2 test (Fisher exact test 2-tailed); **** *t*-test (exact sig 2-tailed).

## Appendix B

**Table A2:** Improvement in patient-experience measures over time (T0 vs. T2) and between control (CG) vs. intervention (IG) groups.

Variables	Total HTx Patients (N = 134)	CG (N = 63)	IG (N = 71)	Statistics OR (IC 95%)	*P*-Value
**Number of patients feeling that they take excessive medication (Yes), N (%)**					
• T0	82 (63)	35 (56)	47 (69)	1.79 (0.9;3.7)	0.109 *
• T2	47 (39)	23 (42)	24 (36)	0.80 (0.4;1.7)	0.540 *
• Statistics OR (IC 95%)	-	3.9 (1.2;12.6)	4.5 (1.2;17.7)		
• *P*-value McNemar test	-	0.167	**<0.001**		
**Degree of inconvenience perceived by the patient related to taking medication as prescribed every day (scored 0-10), M ± SD**					
• T0	2 ± 3	2 ± 2	3 ± 3	-	0.661 *
• T2	1 ± 2	2 ± 3	0.5 ± 2	-	**0.002 ***
• Statistics OR (IC 95%)	-	-	-		
• *P*-value T-test	-	**0.029**	1.94		
**Patients’ awareness of the importance of immunosuppressive therapy and consequences of not taking it*,* N (%)**					
**1. ** **“If you discontinued taking your immunosuppressants completely, what do you think would happen to you? (answer 3: rejection)”**					
• T0	95 (72)	47 (75)	48 (70)	-	0.762 *
• T2	119 (98)	53 (96)	66 (100)	-	0.361
• Statistics OR (IC 95%)	-				
• *P*-value Friedman test	-	**0.001**	**<0.001**		
**2. ** **“If you sometimes forgot to take your immunosuppressants, what do you think would happen to you?” (answer 3: rejection)**					
• T0	77 (59)	41 (66)	36 (52)	-	0.114 *
• T2	107 (88)	47 (86)	60 (91)	-	0.201 *
• Statistics OR (95% CI)	-				
• *P*-value Friedman test	-	**0.012**	**<0.001**		
**Knowledge of the medication regimen (% of medications of total prescribed), M ± SD**					
**Proportion of medication names remembered**					
• T0	76 ± 29	73 ± 33	79 ± 25	-	0.528 **
• T2	84 ± 27	77 ± 32	91 ± 20	-	**0.006 ****
• *P*-value Wilcoxon test	-	0.750	0.197		
**Proportion of medication doses remembered**					
• T0	51 ± 29	51 ± 32	50 ± 26	-	0.864 **
• T2	63 ± 29	56 ± 32	69 ± 25	-	**0.030 ****
• *P*-value Wilcoxon test	-	0.842	0.072		
**Proportion of medication timing intakes remembered**					
• T0	79 ± 25	81 ± 26	79 ± 25	-	0.533 **
• T2	91 ± 21	87 ± 24	93 ± 18	-	**0.019 ****
• *P*-value Wilcoxon test	-	0.792	0.058		
**Proportion of medication indications remembered (the use of that drug for treating)**					
• T0	62 ± 34	58 ± 35	65 ± 34	-	0.213 **
• T2	83 ± 24	77 ± 26	88 ± 22	-	**0.003 ****
• *P*-value Wilcoxon test	-	0.284	**0.014**		
**Number of medication side effects reported by patients, M ± SD, IQR**					
• T0	6 ± 3, 4;7;8	6 ± 3	7 ± 3	-	0.294 **
• T2	3 ± 2, 2;3;5	3 ± 2	3 ± 2	-	0.799 **
• *P*-value T-test	-	**<0.001**	**<0.001**		

See variables and methods section for definitions. See the statistical analysis section in the manuscript for more detail. Missing values were not imputed nor were anomalous values substituted. Measurement points: *T*0 (baseline at inclusion into study), *T*2 (at least after 12 months from inclusion). Abbreviations: HTx, heart transplantation; M, mean; SD, standard deviation; GI, RCT intervention group; CG, RCT control group; OR, odds ratio; RCT, randomized controlled trial. * Pearson’s Chi-squared test (χ2); ** Wilcoxon test.
