# Supplementary material for: Mobile Health to Improve Adherence and Patient Experience in Heart Transplantation Recipients: The mHeart Trial
Source: Healthcare (Basel). 2021 Apr 14;9(4):463. doi: 10.3390/healthcare9040463 (PMC8070926; doi:10.3390/healthcare9040463)
Supplement: Supplementary file 1 [file healthcare-09-00463-s001.pdf]

Multimedia Appendix 1. Description of the mHeart strategy: (A) the intervention design; (B) the intervention workflow; (C) the mHeart® features.

The description of mHeart-based treatment designed to improve medication adherence (the mHeart strategy) has been published in a pilot study performed to validated the feasibility of the intervention and satisfaction of patients with the tool. [1]

The information provided in Multimedia Appendix 1 complements the data provided in the manuscript. The information has been presented according to the Directions for the International Society for Research on Internet Interventions (ISRII) [2] and the CONSORT-EHEALTH guidelines [3] were followed to report the Internet-based intervention.

A) The study intervention description.

The mHeart strategy definition.

- The mHeart strategy designed consisted of an intensive follow-up program based on multilevel individually-tailored digital interventions aiming to change behavior by a pharmacist using the mHeart technology in an interdisciplinary environment. The mHeart intervention focused on increasing the opportunities for professional-recipient interactions, and to enhance patient self-empowerment.

Type of treatment

- The e-interventions were interactive with additional human support through the mHeart platform.
- The provider was a female clinical pharmacist with experience in motivational interviewing and specialized in the heart transplant population. The patients' first interaction with this provider was during hospitalization for the transplant procedure. No other contact was provided on-site after the first baseline study visit.
- The patients' characteristics are described in the manuscript.

The delivery platform: the mHeart tool.

- The hardware platform delivers the intervention via mobile platforms such as smartphones. Patients had access to a complementary website via desktop computers. Providers manage the platform through the website.
- Participants used their own cell phone and paid for their internet use. No incentives were provided for participation.
- The mHeart software (mobile application and website) is a Behavior Intervention Technology to facilitate the following overall goals: (i) health behavior change (i.e. increase patients' healthy behaviors and prevent the onset of disease); and (ii) targeted disease management (i.e. facilitate therapeutic interventions and improve patients' self-management).
- The features specifically designed to manage medication adherence are provided in section (C) of this Multimedia Appendix. Other components or functionalities are detailed in a video of the mobile application provided in [Mendeley Dataset]. [4]
- More information on developers, technical specifications and Source Code are also provided in [Mendeley Dataset]. [4]

Presentation strategy

- The mHeart platform is based on visual aids and minimizing text and passive information. For readers to have a clear sense of the aesthetics, visual aids used and other features, they were provided with a video with a demo trial of the clinical use of the app. Thus, readers can examine samples or portions of eHealth interventions through mHeart.
- Interactive elements were also used as digital triggers to prevent the law of attrition; i.e. (A) alerts, (P) prompts and reminders, (N) notifications, (M) messages, (L) logs, (R) reports, (V) visualizations, (C) video-calls, (I) information delivery. [5,6]

Content

- The interventional treatment design was based on published literature on internet-based interventions with impact on health behavior change, but also strategies to prevent patient attrition. [2,3,7–12]
- All the behavioral change techniques [13,14] used in the treatment are described based on Michie's taxonomy [15] and are provided in the Table. The most important strategies applied were human support, motivational engagement, therapeutic alliance strategies, [5,16] and individually-tailored feedback. [13,14,17–19] Descriptions of the strategies and examples are provided in the Table.
- The strategy could be aimed at (i) forming a behavior; (ii) altering a behavior (iii) reinforcing a behavior.
- The interventions were tailored based on mHeart patient-reported data collected using (i) dynamic information from the mHeart features, and (ii) information collected in the in-clinic baseline interviews.
- Interventions were delivered using motivational interviewing skills. [20,21]

B) The study intervention workflow.

Patient activation in the mHeart software, training and technical assistance received.

At the end of the baseline interview after allocation was known (T0), intervention participants were asked to undergo an initial mHeart training session for 30 minutes in order to: (i) sign the data protection agreement form to use the mHeart platform, (ii) receive verbal and written information about how to set-up and use the mHeart application and website, (iii) receive the mHeart username and code (by a private automated message sent to the patient's phone) after the pharmacist had activated their profile on the mHeart platform, (iv) agree on the scheduled duties of the participant on the mHeart platform according to comorbidities, comedications, and a previous medication management interview.

During the following day, an initial technical mHeart set-up was provided by the mHeart help center of the private firm developing the technology. This session was conducted by telephone and lasted at least 15 minutes to enable at-home monitoring, i.e. (i) downloading the app from the online store, (ii) guiding the first access, and (iii) providing training on the functionalities of the mHeart platform. This service was also responsible for query resolution and user-assistance throughout the study period. When patients had received the telephone training, the transplant pharmacist sent them a welcome message through mHeart requesting a response to confirm their activation in mHeart follow-up. Once the patients had responded to this message, they were considered activated in the mHeart online follow-up.

Specific behavior change techniques selected for use during the theory-based intervention program (the mHeart strategy) and workflow adapted for delivery using the mHeart platform.[1]

- Intended doses and optimal timing for the use of each technique are described in the table.

- Conditions of use, a mixture of time-based, event-based or task-completion rules were applied as required. [22] Thus, the complexity of the strategies varied depending on the patient and the task. A combination of these techniques was common.
- Video calls were not scheduled and were limited to very occasional situations when a text message was insufficient to deliver highly complex information.

Technique (theoretical framework[a])	Description of the behavioral intervention technique and the *element* [b] to support the strategy	Timing[c]	Dose frequency[d]
Motivational communication skills (MI)	Use of any patient-professional communication opportunity to prompt the patient to provide self-motivating statements and self-evaluations: (1) minimize resistance to change; (2) maintain the change achieved. *Element: I,N, M, R, V, C.*	(1),(2)<br>Adh. & Nonadh.	(1),(2)<br>Continuously
Tailoring	Use any opportunity to individualize the management of adherence to a specific patient, based on the patient's environment and self-reports. *Element: all.*	Adh. & Nonadh.	Continuously
Provide instructions (SCogT)	Tell the patient how to adopt a medication-related behavior, i.e. (1) education on the importance of taking immunosuppressive medication and management of side effects. *Element: I, M, C*; (2) responses to the patient's queries and doubts. *Element: M*; (3) information about the prescription change (doses, drug, etc.) and explaining the reason for the change. *Element: M,C, N.*	(1),(2),(3)<br>Adh. & Nonadh.	(1)<br>Baseline<br>& If needed<br>(2),(3)<br>Continuously
Time management	(1) Find a time for intake that fits with each patient's lifestyle. Include this schedule in the patient's mHeart agenda and activate intake alarms if necessary. (2) Train him/her on what to do if intake is late. *Element: A, M, I*	(1),(2)<br>Adh. & Nonadh.	(1),(2)<br>Baseline<br>& If needed
Goal setting (CT)	Involve the professional and recipients in detailed planning of the steps the patient will take to acquire the medication skills needed for adequate medication adherence (frequency, intensity, duration and context). *Element: all.*	Adh. & Nonadh.	Baseline<br>& If needed
Provide information on the behavior-health link (IMB)	Consider providing general information by mass campaigns about behavioral risk, i.e. (1) importance of taking immunosuppressive drugs on time, (2) reminding patients about sun protection adherence. *Element: M*	(1),(2)<br>Adh. & Nonadh.	(1)<br>Baseline & W2<br>(2) W2
Provide information on the consequences (CT)	Inform the patient of the benefits and costs of changing or not changing a behavior (i.e. adherence or nonadherence to medications or monitoring). E.g. pressure rates on range if adhere to antihypertensives. *Elements: M, C, I, R, V*	Adh. & Nonadh.	Once/week & If needed
Prompt self-monitoring of behavior (CT)	Prompt patient to report data related to medication behavior, i.e. (1) drug intake in the agenda*;* (2) medication adherence ePROMs; (3) side effects; (4) glycemia, blood pressure, etc. *Elements: L, M, V, R, P, N*	(2),(3),(4)<br>Adh. & Nonadh.<br>(1) Nonadh.	(1),(2),(4)<br>Continuously<br>(3) If needed
Provide feedback on performance (CT)	Provide the patient with data based on the self-reported information to maintain patient motivation and adherence with the intervention program, i.e. (1) biomeasures pattern; (2) side effects; (3) medication adherence ePROMs; (4) medication intake. *Elements: P, R, V, M*	(1),(2),(3) & (4)<br>Adh. & Nonadh.	(1),(4)<br>Once/week<br>(2)<br>If needed<br>(3)<br>Continuously
Provide contingent rewards (OC)	Provide praise or encouragement linked to the achievement of specified behaviors, e.g. praise any improvement in self-management. *Elements: P, R, V, M*	Adh. & Nonadh.	Continuously
Prompt review of behavioral goals (CT)	Review the intentions or goals previously agreed with the patient; discuss and readjust the plan if necessary. *Elements: M, C*	Nonadh.	Once/week<br>& If needed

Continued on next page è

Technique (theoretical framework[a])	Description of the behavioral intervention technique and the *element* [b] to support the strategy	Timing[c]	Dose frequency[d]
Identify barriers to behavior (SCogT)	Identify the barriers to adequate adherence using PROMs, e.g. detect a specific side effect reported by the patient electronically. *Elements: R*	Nonadh.	If needed
Action planning & problem solving	Plan ways of overcoming the barriers detected and reach an agreement with the patient, e.g. discuss medication beliefs with the patient. *Elements: all*	Nonadh.	If needed
Environmental restructuring	Provide guidance to change the patient's habits that could hamper medication adherence. *Elements: all*	Nonadh.	If needed
Teaching the use of prompts/cues (OC)	Teach the patient to identify environmental cues to remind him/her to adopt a behavior, i.e. (1) times of meals could serve as reminders of medication intake; (2) a beeping signal at the time of scheduled medication intake. *Element: A, P, M*	Nonadh.	Baseline & If needed
Prompt intention formation (TRA,TPB, SCogT, IMB)	Encourage the patient to decide to act or set a general goal, e.g. to make a behavioral resolution such as "I will take my pills on time every day". *Element: I, N, L, M, V*	Nonadh.	If needed
Prompting focus on past success	Discuss or review with the patient past behaviors related to negative outcomes. *Element: M, C, V, R*	Nonadh.	If needed
Others' approval (TRA, TPB, IMB)	Provide information on what others think of a behavior, i.e. inform the patient that professionals will disapprove of an unhealthy behavior. *Element: M*	Continuously Nonadh.	If needed
Provide information on others' behavior	Compare anonymous experiences, e.g., compare the patient's prescription with another significantly more complex regimen to reduce his/her feeling of burden. *Element: M, V*	Nonadh.	If needed
Use follow-up prompts	Communicate the patient if a part of the intervention is complete, i.e. (1) several goals were planned; (2) a behavior has changed gradually. *Element: all*	(1),(2) Nonadh.	If needed
Prompt identification as a role model	Indicate how the patient may be an example to others and influence their behavior, e.g., offer a patient to be part of the voluntary service. *Element: M*	Adh.	W3

[a] The theoretical frameworks are: CT, control theory; IMB, information-motivation-behavioral skills model; MI, Motivational Interview; OC, operant conditioning; SCogT, social-cognitive theory; TPB, theory of planned behavior; TRA, theory of reasoned action.

[b] Elements (i.e. components or objects of the technology intended to implement the strategy) used in the study: (A) alerts, (P) prompts/reminders, (N) notifications, (M) messages, (L) logs, (R) reports, (V) visualizations, (C) video-calls, (I) Information delivery. [5,6]

[c] Nonadherence to medication in the implementation phase is defined as "actual dosing does not correspond to the prescribed dosing regimen due to delays, omissions or extra doses" and is measured by self-report questionnaires. Delays refer to irregularities with the intake schedule (±2 hours).

[d] Definitions: Baseline: when the treatment begins; Continuously: every time the task is scheduled during the treatment period between assessment 1 and 2; If needed: when provider detect that the strategy is needed based on reports or goals established; Once/ week: at least 1 time per week based on reports; W1: during week 1 of the study; W2: during week 2 of the study; W3: during week 3 of the study.

Abbreviations: Adh., medication-adherent recipient; ePROMs, electronic patient-reported measures; Nonadh., only if the patient is classified as nonadherence to medication (implementation phase).

C) The MHeart® features. Patient and professional profiles: modules, components, and clinical use.

Details including videos about how the application works, how the patient interacts with it and a fully description of functionalities have been provided in Mendeley Dataset [4] and have been published in the pilot study.[1]

Patient Module	Components and clinical use
Treatment	Medication list including information on inactive drugs.<br>Enquire about interactions consultation (i.e. ask transplant pharmacist about new therapies).
Patient-Centered Module	Consulting and recording data (manually or using wearables). Reminders can be scheduled in *Agenda*.<br>1. Vital signs (i.e. blood pressure, temperature, pulse and respiratory rate) and biomeasurements (i.e. weight, height, glycemia).<br>2. Dietary intake, exercise data, and general wellness.<br>Health instruments: adherence to medication (Haynes-Sackett [23] and Morisky-Green 4-item scale [24]), insomnia (Insomnia Severity Index [25]) and quality of life (EQ-5D-3L [26]).<br>3. Symptoms or adverse effects. The symptoms connected with an alert to clinicians were diarrhea, vomiting, fever, fainting episode, and syncope.
Agenda	The content of diverse modules is uploaded. A Push text alert can be activated on the patient's mobile phone.<br>1. Medication timing and consultation of recommendations.<br>2. Drug intake recording (single or several drugs at the same time) and reasons for nonadherence (drop-down list).<br>3. Non-pharmacological prescriptions (e.g. relaxation practice according to the psychologist's prescription).<br>4. Tasks from the *Patient-Centered Module* programmed (e.g. blood pressure monitoring 3 times per week).<br>5. Health reminders (e.g., appointments, blood tests).
Communication Aids	1. Teleconference: individual and group sessions.<br>2. A private patient-provider chat. Files can be attached.
Health Advice	Healthy lifestyle and health promotion information (e.g., texts, photographs, or multimedia files).
Personal and Clinical Data	Sociodemographic data, documented allergies and provider profiles (including affiliation and picture).
Help	1. A help center service to solve both technical and functional problems (i.e. telephone number, private message, and email).<br>2. Clinical contact data: medical team, pharmacist, transplant coordinator, patient appointment center, etc.
About	Information about the developers, the aim of the tool, and the team in charge of it.
Terms of Use and Privacy Policy	All the legal requirements already accepted should always be available for consultation.

Provider Module	Component and clinical use
Patient View	List of active patient filters to organize the list and perform a rapid search.
Patient Registration	1. The Center identification number is used to download patient data from the hospital information system.<br>2. The patient receives a private message with login credentials.<br>3. Providers individualizes the patient-reported outcome measures schedule and the treatment plan and recommendations for each new patient.
Treatment Prescription	1. Pharmacological treatment is prescribed from a drop-down list of drugs updated from the Spanish National Formulary. Tailored recommendations can be added (e.g. "Anti-rejection treatment. It is recommended that you take this on an empty stomach").<br>2. Non-pharmacological therapies can be prescribed in free-form data entry by the multidisciplinary team (e.g. non-salty diet).
Patient-Centered Data Consultation	All the data recorded in the *Patient-Centered Module* can be tracked graphically in tables and diagrams. Timeframes filters can be used.<br>mHeart® platform features designed to follow medication adherence are adherence test results and drug intake registrations:<br>1. A traffic light system alerts provider of a decrease in the patient's weekly adherence. List of patients can be sort by adherence rate to prioritize interventions.

	2. Adherence rates are presented graphically and through tables (for each drug and for the overall treatment).
Communication Aids	1. Individual patient-provider chat.<br>2. Group messaging. Filters are available. Large-scale interventions can be scheduled (e.g. preventive health promotions) for specific time periods.<br>3. Teleconsultation patient/s-provider/s for individual or group visit.<br>4. Teleconference for interdisciplinary communication and shared decision-making between providers.

Multimedia Appendix 1 references:

1 Gomis-Pastor M, Roig E, Mirabet S, *et al.* A Mobile App (mHeart) to Detect Medication Nonadherence in the Heart Transplant Population: Validation Study. *JMIR Mhealth Uhealth* 2020;**8**:e15957. doi:10.2196/15957

2 Ritterband LM, Andersson G, Christensen HM, *et al.* Directions for the International Society for Research on Internet Interventions (ISRII). *J Med Internet Res* 2006;**8**:1–6. doi:10.2196/jmir.8.3.e23

3 Eysenbach G, CONSORT-EHEALTH Group. CONSORT-EHEALTH: improving and standardizing evaluation reports of Web-based and mobile health interventions. *J Med Internet Res* 2011;**13**. doi:10.2196/jmir.1923

4 Gomis-Pastor M, Mangues MA, Pellicer V. mHeart - mHealthCare Platform Adapted to the Heart Transplant Population - Technical Specifications and Software Source Code. Mendeley Data. 2019;**V2**. doi:10.17632/yf2dgcfmmh.2

5 Baumel A, Kane JM. Examining Predictors of Real-World User Engagement with Self-Guided eHealth Interventions: Analysis of Mobile Apps and Websites Using a Novel Dataset. *J Med Internet Res* 2018;**20**:e11491. doi:10.2196/11491

6 Muench F, Baumel A. More Than a Text Message: Dismantling Digital Triggers to Curate Behavior Change in Patient-Centered Health Interventions. *J Med Internet Res* 2017;**19**:e147. doi:10.2196/jmir.7463

7 Heron KE, Smyth JM. Ecological Momentary Interventions: Incorporating Mobile Technology Into Psychosocial and Health Behavior Treatments. *Br J Heal Psychol* 2010;**15**:1–39. doi:10.1348/135910709X466063

8 Free C, Phillips G, Galli L, *et al.* The Effectiveness of Mobile-Health Technology-Based Health Behaviour Change or Disease Management Interventions for Health Care Consumers: A Systematic Review. *PLoS Med* 2013;**10**:e1001362. doi:10.1371/journal.pmed.1001362

9 Whittaker R, Merry S, Dorey E, *et al.* A development and evaluation process for mhealth interventions: Examples from New Zealand. *J Health Commun* 2012;**17**:11–21. doi:10.1080/10810730.2011.649103

10 Yen P-Y, Bakken S. Review of health information technology usability study methodologies. *J Am Med Inf Assoc* 2012;**19**:413-422. doi:10.1136/amiajnl-2010-000020

11 Kohl LFM, Crutzen R, De Vries NK. Online prevention aimed at lifestyle behaviors: A systematic review of reviews. *J Med Internet Res* 2013;**15**:1–13. doi:10.2196/jmir.2665

12 Kelders SM, Kok RN, Ossebaard HC, *et al.* Persuasive system design does matter: A systematic review of adherence to web-based interventions. *J Med Internet Res* 2012;**14**:1–24. doi:10.2196/jmir.2104

13 Davis R, Campbell R, Hildon Z, *et al.* Theories of behaviour and behaviour change across the social and behavioural sciences: a scoping review. *Health Psychol Rev* 2015;**9**:323–44. doi:10.1080/17437199.2014.941722

14 Conn VS, Enriquez M, Ruppar TM, *et al.* Meta-analyses of Theory Use in Medication Adherence Intervention Research. *Am J Health Behav* 2016;**40**:155–71. doi:10.5993/AJHB.40.2.1

15 Abraham C, Michie S. A Taxonomy of Behavior Change Techniques Used in Interventions. *Heal Psychol* 2008;**27**:379–87. doi:10.1037/0278-6133.27.3.379

16 Pagès-Puigdemont N, Mangues MA, Masip Giovanna M, *et al.* Patients' Perspective of Medication Adherence in Chronic Conditions: A Qualitative Study. *Adv Ther* 2016;**33**:1740–54. doi:10.1007/s12325-016-0394-6

17 Webb TL, Joseph J, Yardley L, *et al.* Using the Internet to promote health behavior change: A systematic review and meta-analysis of the impact of theoretical basis, use of behavior change techniques, and mode of delivery on efficacy. *J Med Internet Res* 2010;**12**:1–18. doi:10.2196/jmir.1376

18 Kok G, Schaalma H, Ruiter RAC, *et al.* Intervention Mapping: A Protocol for Applying Health Psychology Theory to Prevention Programmes. *J Health Psychol* 2004;**9**:85–98. doi:10.1177/1359105304038379

19 Michie S, Johnston M, Francis J, *et al.* From Theory to Intervention: Mapping Theoretically Derived Behavioural Determinants to Behaviour Change Techniques. *Appl Psychol* 2008;**57**:660–80. doi:10.1111/j.1464-0597.2008.00341.x

20 Salvo MC, Cannon-Breland ML. Motivational interviewing for medication adherence. *J Am Pharm Assoc* 2015;**55**:e354–63. doi:10.1331/JAPhA.2015.15532

21 Miller WR, Rose GS. Toward a Theory of Motivational Interviewing MI was not founded on theory. *Am Psychol* 2009;**64**:527–37. doi:10.1037/a0016830.Toward

22 Mohr DC, Schueller SM, Montague E, *et al.* The behavioral intervention technology model: An integrated conceptual and technological framework for ehealth and mhealth interventions. J. Med. Internet Res. 2014;**16**. doi:10.2196/jmir.3077

23 Sackett DL, Haynes RB, Guyatt GH TP. Ayudar a los pacientes a cumplir los tratamientos. *Epidemiol clínica, Cienc básica para la Med clínica 2ª ed Madrid Médica Panamerica, SA* 1994;:249–78.

24 Carbonell Abella C, Guañabens Gay N, Regadera Anechina L, *et al.* Análisis del cumplimiento terapéutico en mujeres con osteoporosis. *Reumatol Clin* 2011;**7**:299–304. doi:10.1016/j.reuma.2010.12.003

25 Fernandez-Mendoza J, Rodriguez-Muñoz A, Vela-Bueno A, *et al.* The Spanish version of the Insomnia Severity Index: A confirmatory factor analysis. *Sleep Med* 2012;**13**:207–10. doi:10.1016/j.sleep.2011.06.019

26 Rabin R, de Charro F. EQ-5D: a measure of health status from the EuroQol Group. *Ann Med* 2001;**33**:337–43.

27 Gomis-Pastor M, Roig Mingell E, Mirabet Perez S, *et al.* Multimorbidity and medication complexity: New challenges in heart transplantation. *Clin Transplant* 2019;:e13682. doi:10.1111/ctr.13682

Multimedia Appendix 2. Study measures collected.

**Demographic data and clinical information**	
**Demographic information**	**Treatment measures**
• Recipient gender (male)	• Immunosuppressive treatment
• Body mass index (kg/m2)	• Total drugs count
• Recipient age at the time of the study (years)	• Patients with polypharmacy (≥ 8 drugs)
• Patients >75 years old	• Patients with polypharmacy (≥ 15 drugs)
• Educational attainment	• Drugs to treat comorbidities
• Employment status	• Over-the-counter medicines
	• Complementary therapies
**Clinical variables transplant related**	**Multimorbidity and use of care levels of the recipients included in the study**
• Recipient age at HTx (years)	• Number of comorbidities Post-HTx[a]
• Time from HTx (years)	• Need or requirement for caregiver
• Urgent HTx	• Lives with someone else
• Heart failure etiology	
• Donor gender (men)	
• Donor age	
• Total ischemia time (min)	
• Mismatch cytomegalovirus (recipient-/donor+)	
• Number of recipients with at least 1 episode of acute cellular rejection episode	
• Number of recipients with at least 1 episode of antibody-mediated rejection	
• Cardiac allograft vasculopathy (CAV) >1	
• Left ventricular ejection fraction (LVEF %)	
**Patients' access to technology and willingness to use mHealth services**	
• Technology availability	
• Internet access on patients' devices	
• Frequency of technology use	
• Internet usage for health-related purposes	
• Initial assessment of the mHealth approach	
• Initial assessment of the mHeart® type of platform	

- Their need for a tutor to hypothetically guide them in the use the platform

Patients engagement (attrition) with the mHeart tool during the study period

- Using mHeart every day (i.e. All messages received by the team were read on time)
- Using mHeart every day but needed to be reminded to use the mHeart platform at least once during the study period
- Not using mHeart every day (and the reason)
- Not using mHeart at the end of the study (and the reason)

Patient-experience measures

- Self-reliance for medication management
- The perceived inconvenience of their medication regimens (scored 1 to 10)
- Feeling of taking excessive medication
- Opinion about the importance of the immunosuppressive treatment and consequences of not taking it
- Knowledge of their regimen
- Reported medication adverse effects

In-clinic personalized interventions by the pharmacist to improve patients' medication management

- To check for interactions
- To recommend a pillbox
- To assess pill count at the next in-clinic appointment
- To contact the primary care physician or the pharmacy office
- To contact the social worker because of financial problems
- To receive a written regimen timetable
- Therapy optimization. Based on previously published suggested interventions according to the therapeutic complexity observed in our HTx population[27]:
    - Simplifying the number of doses per day
    - Reducing frequency, making administration requirements easier
    - Considering non-pharmacologic alternatives
    - Deprescribing chronic treatments or substituting them
    - Avoiding a prescribing cascade

Intensity of the treatment and in-clinic appointments with the clinical pharmacist to perform medication management follow-up at the end of the study

- No need for regular in-clinic appointments with the clinical pharmacist:
    - Discharge from in-clinic visits
    - Discharge with intensive mHeart reminders to track medication adherence
    - Discharge with mHeart reminders to follow lifestyle habits affecting medication regimens
- Need for face-to-face in-clinic appointments with the clinical pharmacist:

- Intensive in-clinic follow-up every 6 months
- Annual in-clinic follow-up to reinforce medication adherence
- Annual in-clinic follow-up for other medication-related issues

[a] The category comorbid disease included all chronic diagnoses besides the principal diagnosis (i.e., HTx status), lasting 1 year or more, requiring ongoing medical attention, and/or limiting activities of daily living according to the Multiple Chronic Conditions Framework of the US Department of Health & Human Services (2010). Comorbidities were coded according to the 10th revision of the International Statistical Classification of Diseases and Related Health Problems (ICD-10).

Multimedia Appendix 3. Donor and receptor clinical data.

Variables	Patients RCT (N=134)
Donor gender (men), N (%)	72 (56)
Donor age, M ± SD	36±14
Total ischemia time (min), M ± SD	187±57
Mismatch CMV (recipient-/donor+), N (%)	20 (15)
Number of HTxR with at least 1 episode of ARE, N (%)	81 (60)
Number of HTxR with at least 1 episode of AMR, N (%)	7 (5)
CAV >1, N (%)	60 (67)
LVEF (%), M ± SD	66±8

See the statistical analysis section in the manuscript for more detail. Missing values were not imputed nor were anomalous values substituted.

Abbreviations: AMR, antibody-mediated rejection; ARE, acute cellular rejection episode (endomyocardial biopsy 1R); BMI, body mass index; CAV, cardiac allograft vasculopathy; CMV, cytomegalovirus; HTx, heart transplantation; HTxR, heart transplant recipients; LVEF, left ventricular ejection fraction; M, mean; SD, standard deviation.

Multimedia Appendix 4. Technology-related data. A) Patients' technology-related usability and preferences at baseline, B) Patients' engagement with the mHeart mobile application at the end of the study.

**A. Patients' technology-related usability and preferences at baseline.**		**Patients RCT (N=134)**
Types of devices owned by patients, N (%)	Computer	97 (73)
	Tablet	60 (45)
	Mobile	131 (98)
Internet access on patients' devices, N (%)	WIFI	18 (13)
	3G or 4G connection	111 (83)
	Does not know/no response	5 (4)
Frequency of technology use, N (%)	Often	87 (65)
	Sometimes	35 (26)
	Never	12 (9)
Internet usage for health-related purposes, N (%)	Often	41 (31)
	Sometimes	43 (32)
	Never	50 (37)
Initial assessment of the mHealth approach for other patients (hypothetical), N (%)	Not very useful	2 (2)
	Useful	91 (68)
	Very useful	40 (30)
	Not yet known until the platform is tested	1 (0.7)
Initial assessment of the mHealth approach for the patient (hypothetical), N (%)	Not very useful	26 (26)
	Useful	57 (58)
	Very useful	16 (16)
	Not yet known until the platform is tested	0 (0)
mHeart® type of platform initial assessment; N (%) (multiple choice)	Interested in using mHeart® mobile app	81 (86)
	Interested in using mHeart® website	63 (67)
Initially requires a tutor to use the platform, N (%)		29 (24)

**B. Patients' engagement with the mHeart mobile application during the study period.**	**Patients using mHeart (RCT IG) (N=71)**
Using mHeart every day (i.e. all messages received by the team were read on time), N (%)	53 (75)
Using mHeart every day but needed to be reminded to use the mHeart platform at least once during the study period, N (%)	7 (10)
Not using mHeart every day because of technical problems, N (%)	2 (3)
Not using mHeart every day because of lack of interest, N (%)	6 (8)
Not using mHeart every day because of lack of technological skills, N (%)	3 (4)
Not using mHeart at all, N (%)	0 (0)

Abbreviations: HTx, heart transplant; IG, Intervention Group; RCT, randomized controlled trial.

Multimedia Appendix 5. Adherence to medication rates.

		Patients RCT (N=134)		
**Variable**		T_0	T_1	T_2
**Immunosuppressive treatment**				
**SMAQ Global (Adh.), N (%)**	Sum of participants answering 1=yes and/or 2=no and/or 3=no and/or 4= no and/or 5= never	38 (29)	55 (45)	81 (67)
**SMAQ 1, N (%)**	"Do you always take your medication at the appropriate time?" (Yes)	79 (63)	69 (68)	97 (85)
**SMAQ 2, N (%)**	"When you feel bad, have you ever discontinued taking your medication?" (Yes)	20 (16)	8 (8)	3 (3)
**SMAQ 3, N (%)**	"Have you ever forgotten to take your medication?" (Yes)	79 (63)	41 (40)	27 (24)
**SMAQ 4, N (%)**	"Have you ever forgotten to take your medication during the weekend?" (Yes)	14 (11)	9 (9)	6 (5)
**SMAQ 5, N (%)**	"In the last week, how many times did you fail to take your prescribed dose?"			
	• Never	111 (83)	110 (89)	102 (84)
	• 1-2 times	22 (16)	11 (9)	13 (11)
	• 3-5 times	1 (0.7)	1 (0.8)	2 (2)
	• 6-10 times	0 (0)	1 (0.8)	3 (3)
	• > 10 times	0 (0)	1 (0.8)	1 (0.8)
**SMAQ 6, M ± SD**	"Since your last visit, how many whole days have gone by in which you did not take your medication?"	0.5±1	0.5±1	0.6±2
**IMTS Global (Adh.), N (%)**	Sum of participants answering "*No*" to questions 1 and 2	59 (44)	71 (58)	98 (82)
**IMTS (1), N (%)**	"Did you modify the immunosuppressant timetable in the last week?"			
	• No	75 (56)	75 (61)	99 (82)
	• > once	58 (43)	48 (39)	22 (18)
	• I don't remember	1 (0.7)	1 (0.8)	0 (0)
**IMTS (2), N (%)**	"Did you modify the immunosuppressant timetable since the last visit?"			
	• No	60 (45)	71 (57)	97 (80)
	• > once	34 (25)	26 (21)	15 (12)
	• > 5 times	39 (29)	26 (21)	8 (7)

	• I don't remember	1 (0.7)	1 (0.8)	1 (0.8)
**BAASIS Global (Adh.), N (%)**	Sum of participants answering "*No*" to questions 1a, 1b, 2, and 3	64 (59)	62 (71)	69 (70)
**BAASIS (1a) Taking dimension, N (%)**	"Do you remember missing a dose of your IM in the past 4 weeks?" (yes)	18 (16)	11 (12)	7 (7)
	• 1 time	13 (68)	6 (46)	4 (50)
	• 2 times	4 (21)	5 (39)	3 (38)
	• 3 times	0 (0)	2 (15)	0 (0)
	• 4 times	0 (0)	0 (0)	1 (13)
	• > 4 times	2 (11)	0 (0)	0 (0)
**BAASIS (1b) Drug holidays, N (%)**	"Do you remember having skipped two or more doses of your IM in a row in the past 4 weeks?" (yes)	3 (10)	3 (19)	2 (13)
	• 1 time	3 (60)	3 (50)	0 (0)
	• 2 times	0 (0)	3 (50)	2 (100)
	• 3 times	1 (20)	0 (0)	0 (0)
	• 4 times	0 (0)	0 (0)	0 (0)
	• > 4 times	1 (20)	0 (0)	0 (0)
**BAASIS (2) Timing dimension, N (%)**	"Do you remember having taken your IM more than 2 hours before or after the prescribed dosing time in the past 4 weeks?" (yes)	36 (32)	22 (24)	28 (28)
	• 1 time	9 (26)	11 (52)	8 (32)
	• 2-3 times	15 (43)	7 (33)	14 (56)
	• 4-5 times	5 (14)	1 (5)	3 (12)
	• Every 2 to 3 days	2 (6)	0 (0)	0 (0)
	• Almost everyday	4 (11)	2 (10)	0 (0)
**BAASIS (3) Reduction of dose, N (%)**	"Have you altered the prescribed amount of your IM during the past 4 weeks without your doctor telling you to do so?" (yes)	1 (0.9)	2 (2)	2 (2)
**BAASIS (4). Persistence, N (%)**	"Have you stopped taking your IM completely in the past 4 weeks without your doctor telling you to do so?" (yes)	1 (0.9)	0 (0)	0 (0)
**Baasis (5). VAS Scale, M ± SD**	Patients' referred overall adherence past 4 weeks (score 0 to 100)	93±14	95±13	95±8
**Co-medication**				
**Haynes Sackett (Adh), N (%)**	"Most patients have difficulty taking all their tablets. Do you have difficulties taking yours?" (No)	93 (69)	104 (85)	110 (91)

Adherence to visits				
**Attending to all visits, N (%)**		133 (99)	123 (92)	121 (90)

Measurement points: *T*0 (baseline at inclusion into study), *T*2 (at least after 12 months from inclusion). Missing values were not imputed nor were anomalous values substituted. See the statistical analysis section in the manuscript.

Abbreviations: Adh., adherence to medication; Basel Assessment of Adherence to Immunosuppressive Medications Scale (BAASIS); GI, RCT intervention group; CG, RCT control group; HTx, heart transplantation; Immunosuppressive Medication (IM); Immunosuppressive Medication Timing Scale (IMTS); M, mean; Nonadh., Nonadherence to medication; OR, Odds Ratio; RCT, Randomized controlled trial; Simplified Medication Adherence Questionnaire (SMAQ); SD, standard deviation; Visual Analog Scale (VAS).

Multimedia Appendix 6. Patient-experience outcomes.

Variable		Patients RCT (N=134) T_0	T_1	T_2
**The patient prepares and takes his/her medication autonomously (Yes), N (%)**		119 (89)	113 (93)	110 (82)
**Person who helps the patient with medication management, N (%)**	Partner	4 (50)	2 (25)	3 (43)
	Children	1 (13)	1 (13)	1 (14)
	Caregiver	1 (13)	2 (25)	2 (29)
	Pharmacy office	0 (0)	0 (0)	0 (0)
	Others	2 (25)	3 (38)	1 (14)
**Number of patient's feeling of taking excessive medication (Yes), N (%)**		82 (63)	53 (43)	47 (39)
**Degree of inconvenience perceived by the patient related to taking his/her medication as prescribed every day (scored 0-10)**	M ± SD	2±3	2±3	1±2
	0-2; 3-6; >7	73 (55); 49 (37); 12 (9)	87 (71); 25 (20); 11 (9)	99 (82); 17 (14); 5 (4)
**Patients' awareness of the importance of immunosuppressive therapy and consequences of non-taking it, N (%)**				
1. "If you discontinued taking your immunosuppressants completely, what do you think would happen to you?"	Nothing	3 (2)	1(0.8)	0 (0)
	I don't know	34 (26)	16 (13)	2 (2)
	A different answer involving rejection	95 (72)	107 (86)	119 (98)
2. "If you sometimes forgot to take your immunosuppressants, what do you think would happen to you?"	Nothing	13 (10)	5 (4)	4 (3)
	I don't know	41 (31)	34 (27)	10 (8)
	A different answer involving rejection	77 (59)	85 (69)	107 (88)
**Knowledge of the therapeutic regimen: % of the number of drugs of the total prescribed, M ± SD**	Proportion of drugs names remembered	76±29	80±30	84±27
	Proportion of drugs doses remembered	51±29	53±30	63±29
	Proportion of drugs intakes remembered	79±25	89±20	91±21

	Proportion of drugs indications remembered	62±3	79±30	83±24
**Number of medication adverse effects reported by patients, M ± SD, IQR**		6±3, 4;7;8	4±3, 2;4;6	3±2, 2;3;5
**Type of medication adverse effects reported by patients, N (%)**	Tremor	79 (62)		
	Skin disorders	71 (55)		
	Visual impairment	61 (48)		
	Emotional lability	63 (49)		
	Cramps	63 (49)		
	Mood swings	60 (47)		
	Tiredness or fatigue	60 (47)		
	Headache	53 (41)		
	Insomnia	44 (34)		

Measurement points: *T*0 (baseline at inclusion into study), *T*1 (at least after 6 months from inclusion), *T*2 (at least after 12 months from inclusion). Missing values were not imputed nor were anomalous values substituted. See the statistical analysis section in the manuscript.

Abbreviations: HTx, heart transplantation; M, mean; RCT, Randomized controlled trial; SD, standard deviation.

Multimedia Appendix 7. Prevalence of in-clinic personalized interventions by the pharmacist to improve patients' medication management.

	Patients RCT (N=134)		
	T_0	T_1	T_2
**Number of patient-centered interventions during on-site visits, M ± SD**	3±1	3±2	2±2
• To recommend a self-managed pillbox, N (%)	81 (60)	28 (23)	24 (20)
• To recommend a pillbox pharmacy office made, N (%)	3 (2)	0 (0)	0 (0)
• To assess pill count at the next in-clinic appointment, N (%)	1 (0.7)	0 (0)	0 (0)
• To contact the primary care physician or the pharmacy office, N (%)	3 (2)	11 (9)	9 (8)
• To contact the social worker because of financial problems, N (%)	0 (0)	1 (0.8)	1 (0.8)
• To receive a written regimen timetable, N (%)	20 (15)	7 (6)	6 (5)
• Optimization interventions to reduce therapeutic complexity, [a] N (%)	94 (71)	75 (62)	47 (39)
• To check for drug-drug, drug-disease or herbal-drug interactions, N (%)	103 (78)	88 (73)	82 (68)

[a] Therapy optimization strategy based on previously published suggested interventions according to the therapeutic complexity observed in our HTx population. (M. Gomis-Pastor et al. *Clinical Transplantation*, 2019)

Measurement points: *T*0 (baseline at inclusion into study), *T*1 (at least after 6 months from inclusion), *T*2 (at least after 12 months from inclusion). Abbreviations: HTx, heart transplantation; M, mean; RCT, Randomized controlled trial; SD, standard deviation.
